# Willingness to use pre-exposure prophylaxis (PrEP) for HIV prevention among men who have sex with men (MSM) in Malaysia: findings from a qualitative study

**DOI:** 10.7448/IAS.20.1.21899

**Published:** 2017-08-02

**Authors:** Adam Bourne, Matteo Cassolato, Clayton Koh Thuan Wei, Bangyuan Wang, Joselyn Pang, Sin How Lim, Iskandar Azwa, Ilias Yee, Gitau Mburu

**Affiliations:** ^a^ Australian Research Centre in Sex, Health & Society, La Trobe University, Melbourne, Australia; ^b^ Sigma Research, London School of Hygiene & Tropical Medicine, London, UK; ^c^ International HIV/AIDS Alliance, Brighton, UK; ^d^ Malaysian AIDS Council, Kuala Lumpur, Malaysia; ^e^ International Program, Australian Federation of AIDS Organisations, Bangko, Thailand; ^f^ Department of Social and Preventive Medicine, University of Malay, Kuala Lumpur, Malaysia; ^g^ Infectious Diseases Unit, Department of Medicine, Faculty Of Medicine, University Of Malaya, Lancaster, Kuala Lumpur, Malaysia; ^h^ Division of Health Research, University of Lancaster, Lancaster, UK

**Keywords:** MSM, PrEP, qualitative, Malaysia, risk reduction, HIV prevention

## Abstract

**Background**: Men who have sex with men (MSM) continue to be disproportionately affected by HIV in Malaysia. Recent success has been observed within demonstration projects examining the efficacy of HIV pre-exposure prophylaxis (PrEP), an antiretroviral -based medication taken by HIV-negative men to prevent sero-conversion. In order for such promising findings to be translated in real-world settings, it is important to understand the acceptability of PrEP, including perceived barriers to access or uptake.

**Methods**: As part of a larger mixed-methods study exploring acceptability and willingness to use PrEP among MSM in Malaysia, 19 men took part in audio-recorded focus group discussions hosted by a community-based HIV organization and facilitated by a trained researcher. Discussions focussed on awareness and potential information management, general perceptions of PrEP and potential motivations or barriers to the use of PrEP, including those at the personal, social, health system or structural level. Data were transcribed verbatim and underwent a detailed thematic analysis.

**Results**: Rather than perceiving PrEP as a replacement for condoms in terms of having safer sex, many participants viewed it as an additional layer protection, serving as a crucial barrier to infection on occasions where condom use was intended, but did not occur. It was also perceived as more valuable to “at-risk” men, such as those in HIV sero-discordant relationships or those with a higher number of sexual partners. Elements of discussion tended to suggest that some men taking PrEP may be subject to stigma from others, on the assumption they may be promiscuous or engage in high-risk sexual behaviours.

**Conclusions**: This qualitative study indicates that, broadly speaking, PrEP may be acceptable to MSM in Malaysia. However, in order for its potential to be realized, and uptake achieved, educative interventions are required to inform the target population as to the efficacy and potential, positive impact of PrEP. Given concerns for how those taking it may be stigmatized, it is crucial that the use of PrEP is presented as a responsible course of action, and one of a range of strategies that men can use to keep themselves safe from HIV.

## Background

Pre-exposure prophylaxis (PrEP) is a significant development in the continuing global efforts to prevent transmission and acquisition of HIV [1,2]. In order to realize the potential of this antiretroviral medication in preventing acquisition of the virus by HIV-negative people, it is crucial that we understand how it is perceived by most-at-risk populations, and assess their willingness to use it. A recent integrated bio behavioural survey found that 8.9% of men who have sex with men (MSM) in Malaysia were infected with HIV [3]. The survey also showed that HIV prevalence was highest in two major cities: Kuala Lumpur at 22.0% and Johor at 15.7%. Community surveys of MSM in Malaysia have reported a high prevalence of condomless anal intercourse with casual partners [4], and a high usage of drugs before and during sex [5], which is associated with a greater likelihood of HIV transmission [6]. While condoms are known to have an impact on HIV epidemics across the world and have prevented a significant number of new infections [7], they are often inconsistently used [8,9] and have proved insufficient in meeting the sexual health needs of all MSM [10,11].

Within the last few years, the potential for PrEP to significantly reduce the number of new infections has become more clearly established. An open-label randomized trial in the UK and a double-blind randomized control trial in France both reported that MSM taking PrEP were 86% less likely to contract HIV compared to those not taking it [12,13]. With respect of the UK trial, nearly all cases of sero-conversion were among men who were non-adherent to the medication, emphasizing adherence as a key determinant of PrEP efficacy. The World Health Organisation now strongly recommends PrEP provision for MSM as a key population in the ongoing HIV epidemic [14].

However, understanding the perceptions of communities most in need of PrEP is an important first step towards realizing the full potential of PrEP demonstrated in the optimal conditions of clinical trials [12,13]. While not entirely predictive of uptake or continued use, understanding the acceptability of, and willingness to engage with, an intervention is central to its development and scale-up. Such an understanding can help counter or mitigate unintended consequences or potential barriers, and ensure its benefit is communicated in the most effective manner [15,16]. With this in mind, we undertook a qualitative study of PrEP acceptability among MSM living in Malaysia. As part of a larger mixed-method study, the specific aim of the qualitative research component described here was to understand the willingness of MSM to use PrEP, including their broad perceptions of utility, as well as motivators and barriers to its potential uptake and use.

## Methods

This qualitative study comprised of focus group discussions (FGDs), with a sub-sample of participants recruited from a larger sample of MSM who participated in an online survey of PrEP acceptability. In the primary online survey, MSM were recruited from gay geo-social networking applications (apps), and through contacts of MSM and HIV community-based organizations. The eligibility criteria for completing the online survey included being biological male, Malaysian citizen, aged 18 years or above, identifying as a man who has sex with men, and be either HIV negative or unaware of their HIV status. Findings related to the quantitative survey are reported elsewhere [submitted under review].

At the end of the online survey, participants were asked whether they would be interested in taking part in FGDs exploring the acceptability of PrEP in greater depth. The same eligibility criteria applied for this qualitative phase, with the additional requirement that participants must reside in Kuala Lumpur. Those who expressed interest and who were living in Kuala Lumpur were sent additional information about the focus groups, including details on when they would be held. Ultimately, 19 men were able to attend and constituted the final sample of this qualitative study. These 19 men were split across three focus groups, each held in Kuala Lumpur at the offices of a community-based HIV organization and facilitated by a trained researcher (author CKTW). All participants were advised that their prior survey responses would not be linked to their participation in this phase in any way. Four participants declined the invitation to provide a full account of their demographic details although they did allow their race to be recorded. All but one described themselves as gay.

Following the provision of informed consent, demographic data were collected via private self-completed short paper form (displayed in Table 1), and definitions of PrEP distinguishing it from PEP were provided. Audio-recorded discussions were then facilitated with the aid of a topic guide that was designed based on the existing literature and study aims, piloted and revized. Participants were asked to describe their involvement in gay social scenes and community structures possibly providing access to HIV services in Kuala Lumpur. Data were collected on perceptions of PrEP in general, and the potential motivators and barriers to use of PrEP (operating at personal, community, health service and structural levels). Participants were also asked to consider how information about PrEP might best be disseminated to MSM in Malaysia, and where they would prefer to access it.

Audio recordings from FGDs were transcribed and translated verbatim into English. Data were anonymized and pseudonyms given to FGD participants in order to protect their identity. The collated data underwent a thematic analysis, supported by NVIVO 10 [17]. This process involved the reading and re-reading of data to identify initial codes (relevant or significant features) via constant comparative approach [18]. The meaning of these codes was discussed, agreed and defined between GM, AB and CK, following which all sections of transcript were assigned to one or more of these codes until saturation was achieved. Coded data were then organized into higher-level relevant themes through an inductive approach [19], and all examples of each potential theme were documented. Identification of key themes was undertaken by GM and corroborated by AB and CK.

**Table 1.** Participant demographic characteristics

**Characteristic** **Age (years)**	*N*	**Characteristic** **Income**	*N*	**Characteristic** **Education level**	*N*
Mean	30	Not stable	2	Diploma	3
Range	20–44	Less than RM 2000	3	Lower university degree or higher	12
		RM 3001–4000	2		
		RM 4001–5000	3		
		RM 5001–6000	2		
		More than RM 6000	3		
**Employment status**		**Race**		**Currently in relationship with a man**	
Full time	10	Chinese	12	Yes	7
Part time	1	Malay	7	No	8
Self employed	1				
Unemployed	1				
Student	2				

### Ethical considerations

The study was granted ethical approval by the Medical Ethics Committee of University Malaya Medical Centre (MECID No: 20161-2010). Pseudonyms are used alongside quotes from participants.

## Results

First, we present data related to the range of risks that men perceived in their sexual environments – some of which PrEP could potentially help to alleviate, while others were new or exacerbated in the context of potential PrEP use. Second, we explore the assumptions that participants in all groups often made regarding the characteristics of men who would use PrEP, how they would be perceived by others and how their sexual behaviour might change due to PrEP use. Finally, we present data on how participants would like to access both PrEP information and prevention initiatives.

### Relative risks and protection

While, in the main, participants recognized how PrEP may reduce the risk of HIV acquisition for a HIV-negative man, they frequently positioned such risk reduction within the context of other prevention strategies and the additional risks that needed to be considered when having sex. Many participants commented that PrEP might provide an additional “layer” of protection, over and above that offered by condoms. Several accounts were given of problems using condoms previously, prior difficulties with negotiating condom use with sexual partners, and a discourse of being “carried away with the moment” and not remembering to use them. In the context of a continuing threat from other sexually transmitted infections (STIs) that several participants articulated, perceptions were held that PrEP offered an additional sense of security on those occasions when condoms were not used as intended.

I think that by using condoms next to PrEP, it is another layer of protection. So, by breaking down one layer, you still have something to prevent [HIV infection] from happening. [Gil]

Several participants in two of the focus groups discussed future hypothetical situations in which they might wish to stop taking PrEP, such as if they became less sexually active, or if they were in a relationship with a partner whom they trusted and who they perceived to be HIV sero-concordant.

However, a number of potential challenges – or risks – to the effective use of PrEP were discussed, which influenced men’s hypothetical or notional willingness to take it. These included the concern of a lack of discipline to take PrEP on a daily basis, thus rendering it ineffective in preventing HIV acquisition. Condoms were often preferred to PrEP by participants because they had been used for a longer period of time and they also represented a visible physical barrier, as one of the participants put it as follows:

There is a physical barrier that we can see in the condom, rather than drugs. [Johnson]

Others simply indicated that they did not perceive a sufficient risk in their sexual lives to need PrEP themselves. They perceived current prevention measures such as their consistent use of condoms, or maintenance of a monogamous relationship, as sufficient. However, several of these men also expressed that they would consider PrEP in the future if they have a higher number of concurrent sexual partners. There was no agreement in the group as to what might constitute a “higher number”.

The risk of potential side effects was another oft-cited reason for the reluctance to start PrEP. Participants cited fear of nausea, feeling poorly, mood swings and damage to the liver, kidneys or other organs as symptoms they perceived to be features of HIV medication and/or those they had observed in HIV-positive friends. Several men across all three focus groups also expressed concern about the clinical effectiveness of PrEP among Malaysian or other South East Asian men. When hearing that the evidence was largely drawn from European, North American and African samples, these participants voiced concern that PrEP may not work for Asian men.

[Test subjects] are not Asians, not Malaysians. So that would still reduce my confidence with regard to taking the pill. [Tim]

The risk of confidentiality being breached during PrEP access was raised in two of the focus groups. In an environment where MSM were criminalized, data protection of MSM’s identities was highlighted as significant in constraining willingness to use PrEP. Participants were concerned that Malaysian National Health Information Systems and Registers, which are widely used in government hospitals, would disclose their sexual identity and deter PrEP users from accessing government health services, as one of the participants expressed:

In Malaysia, MSM is not in our culture yet. In the government clinic you have to register, it has to be on record, so that*’*s not discreet. [Vincent]

### Assumptions and stigma

Participants frequently made assumptions about the “type” of man who might take PrEP, how they might be perceived by others and how PrEP use might influence wider aspects of their sexual behaviour. While the men taking part in these focus groups stated they would be supportive of men who use PrEP, perceiving them as taking responsibility for their own and their sexual partners’ health, they also recognized that *other* MSM may perceive those who use PrEP negatively. Some participants felt that being on PrEP in Malaysia would be perceived by others as having a direct association with riskier behaviours such as *barebacking* or “raw sex” and using recreational drugs for sexual pleasure (colloquially known as “Chem Fun”): *[A PrEP user is someone who] has a lot of unprotected sex (Effan)]*. For one of the participants, using PrEP was also directly associated with sex work where one may not be able to consistently use condoms with clients:

[A PrEP user is] a money boy or go-go boy who is not in a position to negotiate safe sex. [Lik Teng]

Another of the key concerns articulated by participants related to compensatory risk-taking while using PrEP. Several men felt that PrEP could be used as a “party drug”, serving to increase sex (in general) and sexual risk-taking specifically:

People might abuse it and use it like a party drug and a reason to have unprotected sex. So I think there are two sides of the coin. [Gil]

Participants shared a fear that PrEP could be misrepresented as a “magic pill”, which would be associated with lowering inhibitions and “allowing” riskier sexual encounters among MSM. However, one respondent narrated how he changed his mind about PrEP: at first he thought that PrEP would promote riskier sexual behaviours, but with more information about it he later realized that it could be used as an effective HIV prevention method:

At first I was actually against PrEP […] because people will just go ahead and have unprotected sex […] But when I started to know more about it, I think that it’s better to do that because it’s preventive. [Vincent]

### PrEP information and access needs

Participants were regularly using online gay platforms to meet up and to access information in relation to HIV and generally felt that this would be the best space in which to provide information or education around PrEP. They expressed that a well-designed website inspired more confidence, and appeared to be more trustworthy. They viewed some of the local Malaysian websites as “over-simplified” or biased in the way information was presented. In contrast, international websites (such as those of the Mayo Clinic or US Centers for Disease Control were perceived to provide more credible information in relation to PrEP, partly because participants believed that the content of these was more likely to have undergone rigorous evidence checking.

We don’t want to know that Truvada works, we want to know ‘how’ Truvada works […] We want to know how it works and so we want to know more details about HIV prevention and everything in detail […] Outside sources tend to be more credible [than Malaysian ones]. [Johnson]

Overall, participants expressed a strong preference for PrEP to be available online, via specialist applications or websites, which could assess their needs for PrEP and arrange home delivery, offering both convenience and anonymity. In relation to physical access locations, diverse views were expressed. Some felt that the Malaysian government should provide free access to PrEP, while others argued that it should be offered through the public health service or through pharmacies. The potential cost of PrEP, if they had to buy it, was perceived as a deterrent to using it, and influenced where participants though they might access it. They expressed that PrEP should be free of charge at the point of access, especially in government facilities, or cost no more than RM 50–200 (USD 12–49) per user per month, to encourage its use. While some participants were concerned that non-governmental organizations may not have sufficiently trained medical staff to dispense the medication, others believed that their proximity and connection to the communities of MSM made them more accessible.

## Discussion

Efficacy of PrEP has been demonstrated from other studies, and findings from this qualitative study suggest that, broadly speaking, it may be acceptable to MSM in Malaysia. Most participants acknowledged the role that it could play in minimizing HIV acquisition in their context. Rather than seeing PrEP as a replacement for condoms, a majority of participants felt that PrEP could provide a valuable extra layer of protection for those occasions when condom use was intended, but did not occur. The participants described several different sub-populations of MSM who they felt might benefit the most from PrEP, including men who sell sex as well as those who engage in group sex or sex under the influence of drugs. While such men may indeed be good candidates for PrEP, there are many others who are at substantially high risk of acquiring HIV from regular sexual exposures, who may benefit from it. However, evidence from multiple countries over the last two decades has shown that many MSM often struggle to accurately perceive their personal risks for contracting HIV, particularly when entering into new romantic relationships [20,21]. Advice and guidance from community-based HIV prevention programmes and sexual health clinics around the relative risk of acquiring HIV will be central to optimal PrEP utilization.

After 30 years of a HIV prevention response that has relied upon condoms as the most effective and available strategy, it should not be surprising that some MSM will associate safer sex only with their use. While PrEP has been shown to protect from HIV, it does not provide a visible separation of bodily fluids, which as research relating to HIV treatment as prevention indicates, is sometimes perceived as offering greater certainty of preventing transmission [11]. PrEP is still a relatively new method and levels of community awareness and understanding are likely to be low. Longitudinal observation of PrEP rollout would enable the assessment of if, and how conceptions of safer sex change over time with the advent of this new prevention method.

The potential social stigma of PrEP use described in this study mirrors concerns expressed elsewhere about “slut-shaming” [22]: the possibility (or indeed reality) that those who use PrEP are considered promiscuous or otherwise risky, or else they would not need to take the medication in the first place. The ongoing stigmatization of behaviours that warrant the use of PrEP [23] can potentially prevent those at risk of HIV from taking proactive steps to protect their sexual health. In the roll-out of PrEP, it will be crucial to educate the wider populations as to the purpose and efficacy of PrEP to ensure that such stigma does not operate as a barrier to effective uptake and use. In the context of Malaysia, this will require positioning of PrEP as HIV prevention strategy for all populations at substantial risk, including sex workers, MSM, transgender people, people who use drugs and sero-discordant couples, such that MSM are not singled out as the only group that are at high risk of HIV.

These qualitative findings from Malaysia reinforce those observed in other countries, particularly with respect of a concern for PrEP side effects and the negative way in which users may be perceived by others [24,25]. There was also some concern regarding the need for long-term adherence, which has been observed in other settings [25]. Although current regimens propose daily dosing of PrEP in the form of a tablet to build up sufficient protective efficacy, evidence is also emerging that PrEP can be taken on a more intermittent basis and still protect the individual from HIV infection [12]. The feasibility of such intermittent dosing strategies will need to be evaluated in different contexts.

An important unique finding, over and above the generation of findings within this specific cultural and social setting, is the manner in which participants queried the effectiveness of PrEP for Asian men. Concern was expressed that studies of clinical effectiveness in Caucasians may not be replicable in Malaysia due to perceived variations in the physiology of Asian men. Such a concern for the broader applicability of clinical findings to Asian populations, while likely unfounded, highlights the perceived importance of locally situating research findings, taking into account prevailing cultural norms or beliefs. While further clinical efficacy data specific to Asian MSM is likely unwarranted, further implementation science research that helps to target and tailor PrEP information to men in Malaysia may be needed to counter prevalent misconceptions.

As the HIV epidemic in Malaysia shifts from one that was traditionally driven by injecting drug use to sexual transmission (especially between men) [3,26], PrEP could play a significant role in reducing incidence. Recognizing the emerging evidence of effectiveness, PrEP has been included as a preventive tool to mitigate sexual transmission of HIV in specific key populations including MSM in Malaysia’s 3rd National Strategic Plan to end AIDS (2016–2030, 27). A national PrEP consultation with various stakeholders was undertaken in May 2016 whereby Ministry of Health representatives were largely supportive of PrEP but made key recommendations on the need for more operational research and demonstration projects to study the feasibility of PrEP implementation in key populations. In the interim period, PrEP is currently available through a small number of private clinics and one HIV treatment centre in a university teaching hospital in Kuala Lumpur. Following the National PrEP Consultation, The Malaysian HIV Association released the first National Guidelines on PrEP as part of the Updated Consensus Guidelines on Antiretroviral Therapy 2016 [28]. These guidelines currently make no mention of the potential negative impact of stigma in the uptake and maintenance of PrEP use, and do not consider the potential role of online assessment and delivery, a stated preference of the men taking part in this study. Future research and guidelines may wish to consider interventions that address each of these concerns.

It is important to note limitations of this study. As a relatively small, self-selecting sample of MSM from a major urban area who were motivated to discuss PrEP in a focus group environment participated in this study, caution must be taken in applying these findings to all sections of the MSM population. In addition, this study focuses on men’s willingness to use PrEP in a hypothetical scenario. At the time data was collected, PrEP was not widely accessible in Malaysia and prior research has documented differences in perceived acceptability or willingness to engage with medications depending on whether they are perceived as real and accessible, or whether personal use is still hypothetical [29,30]. While willingness is not always a good indicator of future use, qualitative examination such as this helps to identify misinformation, cultural values and beliefs, and concerns that need to be addressed to adequately counsel those at highest risk for HIV who would benefit from PrEP use. Further to this, our sample had a relatively good awareness of PrEP, having participated in a previous online PrEP survey, and therefore may not be similar to all MSMs in Malaysia. Awareness of PrEP is a critical determinant of willingness and acceptability to use it [31], and therefore public health implementation will require heavy focus on effective information dissemination and promotion of PrEP.

## Conclusions

Given increasing evidence related to willingness to use PrEP among self-selected MSM from this and other studies [32–34], it is critical to move to the next phase of site assessments and demonstration of PrEP provision in low- and middle-income countries, including Malaysia. Within this, it will be important to mitigate factors that prevent access to PrEP, such as low awareness of PrEP, fear of side effects, poor HIV risk perception, cost and concerns regarding long-term adherence, while accentuating facilitators of its access and utilization, such as friendly and non-stigmatizing health services and attitudes, motivation to remain healthy, and cost-free provision, among others, depending on context.
